# Aberrant Structure MRI in Parkinson’s Disease and Comorbidity with Depression Based on Multinomial Tensor Regression Analysis

**DOI:** 10.3390/jpm12010089

**Published:** 2022-01-11

**Authors:** Xuan Cao, Fang Yang, Jingyi Zheng, Xiao Wang, Qingling Huang

**Affiliations:** 1 Division of Statistics and Data Science, Department of Mathematical Sciences, University of Cincinnati, Cincinnati, OH 45241, USA; xuan.cao@uc.edu (X.C.); njualicia@gmail.com (F.Y.); 2 Department of Mathematics and Statistics, Auburn University, Auburn, AL 36849, USA; jingyi.zheng@auburn.edu; 3 Department of Radiology, Affiliated Brain Hospital of Nanjing Medical University, Nanjing 210019, China; cx_nju@163.com

**Keywords:** Parkinson’s disease, depression, mood disorders, MRI, structural MRI, diagnosis, prognosis, tensor regression, multinomial regression, gradient descent

## Abstract

Background: Depression is a prominent and highly prevalent nonmotor feature in patients with Parkinson’s disease (PD). The neural and pathophysiologic mechanisms of PD with depression (DPD) remain unclear. The current diagnosis of DPD largely depends on clinical evaluation. Methods: We proposed a new family of multinomial tensor regressions that leveraged whole-brain structural magnetic resonance imaging (MRI) data to discriminate among 196 non-depressed PD (NDPD) patients, 84 DPD patients, 200 healthy controls (HC), and to assess the special brain microstructures in NDPD and DPD. The method of maximum likelihood estimation coupled with state-of-art gradient descent algorithms was used to predict the individual diagnosis of PD and the development of DPD in PD patients. Results: The results reveal that the proposed efficient approach not only achieved a high prediction accuracy (0.94) with a multi-class AUC (0.98) for distinguishing between NDPD, DPD, and HC on the testing set but also located the most discriminative regions for NDPD and DPD, including cortical regions, the cerebellum, the brainstem, the bilateral basal ganglia, and the thalamus and limbic regions. Conclusions: The proposed imaging technique based on tensor regression performs well without any prior feature information, facilitates a deeper understanding into the abnormalities in DPD and PD, and plays an essential role in the statistical analysis of high-dimensional complex MRI imaging data to support the radiological diagnosis of comorbidity of depression with PD.

## 1. Introduction

Parkinson’s disease (PD) is a major neurodegenerative disease influenced by both genetic and environmental factors [1]. As the second most common neurodegenerative disorder, PD is characterized by the degeneration of dopamine-producing cells in the brain, presenting a broad range of symptoms from motor dysfunctions to nonmotor psychobehavioral manifestations [2,3].

Nonmotor features can appear in the earliest phase of the disease even before clinical motor impairment [4,5,6]. Depression is a prominent nonmotor feature which is highly prevalent early in the disease process and has a significant impact on quality of life and disability [7,8,9]. Although common in other chronic diseases [10,11,12], research suggests that depression and anxiety are even more common in PD. It is generally accepted that clinically significant depressive disturbances occur in 40–50% of patients with PD [13]. As such, depression is one of the most frequently reported neuropsychiatric disturbances in PD and influences many other clinical aspects of the disease [14]. In addition to causing inherent emotional distress, depressive disorders negatively impact quality of life, motor and cognitive deficits, functional disability, and other psychiatric comorbidities in patients with PD [14]. Knowledge of the pathophysiology of PD depression remains limited, and available diagnostic tools are better at detecting motor symptoms than nonmotor symptoms, such as depression [15]. Clearly, physician recognition and treatment in PD with depression (DPD) is not enough. DPD on self-report was not recognized by more than 60% by neurologists according to the Unified Parkinson’s Disease Rating Scale (UPDRS) [8], while a large sample study of 1449 outpatients with PD revealed that depression rates were already substantially elevated at an early PD stage [16].

Although the neural and pathophysiologic mechanisms predicting rates of DPD progression remain unclear and are key research priorities. Understanding the inner working mechanisms and discovering biomarkers of DPD is one of the most intriguing scientific questions. Studies in neuroscience strongly suggest intervention during early therapeutic windows [6,17]. PD is a model candidate for precision-medicine-based approaches, which customizes treatments based on patients’ individual genotype and may help reach disease modification [18,19]. Clinical trials have been underway that target specific PD risk genes and their protein products [18,20]. The National Institute of Mental Health Research Domain Criteria (RDoC) initiative grew out of the agency’s goal to develop new ways of classifying mental disorders based on behavioral dimensions and neurobiological measures and efforts have been devoted to understand depression within the context of RDoC by seeking an integrative understanding of the disorder across multiple units of analysis from genes to neural circuits to behavior [21,22].

A variety of neuroimaging technologies, including functional magnetic resonance imaging (fMRI), structure MRI (sMRI), positron emission tomography (PET), and electroencephalography (EEG), have also been adopted for PD diagnosis. The recent Movement Disorders Society Clinical Diagnostic Criteria for PD have included the results of a few of these neuroimaging techniques to serve as single supportive criteria or absolute exclusion criteria for the diagnosis of PD [23]. Structural MRI and advanced MR techniques have been used for the classification of PD and the atypical Parkinsonian syndromes. Thus, leveraging neuroimaging techniques may lead to an early, accurate, and objective diagnostic classification by highlighting the underlying neurochemical and neuroanatomical changes that underlie this spectrum of disorders [24,25].

Structure MRI has received more research focus with better stability and repeatability compared to fMRI, where there were concerns about accuracy due to noise [26,27]. A brain microstructural study found decreased white matter fiber characteristic in right arcuate fasciculus and bilateral middle cerebellar peduncles and increased network connectivity in prodromal early PD, which might indicate neural compensation [28]. A diffusion tensor imaging (DTI) study of white matter microstructure changes found that FA in the mediodorsal thalamus decreased, and there was a relationship between FA in the mediodorsal thalamus and DPD. Another microstructure difference was found between the DPD and non-depressed PD (NDPD) in the bilateral mediodorsal thalamic regions, but the sample size was relatively small and the clinical score included only the Hamilton depression rating scale (HAMD) [29].

Machine learning and artificial intelligence are recognized as booming and promising methods used to detect connectivity [30]. A study of network abnormalities among non-manifesting PD related to gene Leucine Rich Repeat Kinase 2 (LRRK2) mutation carriers displayed significant non-motor cerebral changes among populations “at risk” for future development of PD [27]. More recently, a computer-based technique utilizing convolutional neural networks (CNN) [31,32,33] to create prognostic and diagnostic biomarkers has generated a lot of attention. However, these methods typically require significantly large memory and extensive computation time. In addition, the intuitions behind these machine learning methods are not apparent as the model parameters could not be explicitly interpreted. On the contrary, the tensor regression model [34,35] is a regression framework that treats clinical outcomes as response and images as covariates in the form of multi-dimensional arrays. These tensor regression methods could not only resolve the computational and modeling challenges of large-scale imaging data but could also achieve perfect accuracy, even in smaller sample sizes.

Up until now, most of the existing methods have focused on either the individual diagnosis of PD or the progression of depression comorbidity without simultaneously inferring the onset as well as the stage of PD. In this study, our goal was to build and validate a multinomial tensor-regression-based framework that leveraged three-dimensional (3D) sMRI data to differentiate between non-depressed PD, depressed PD, and healthy subjects. We used the method of maximum likelihood estimation coupled with state-of-art gradient descent algorithms to predict the individual diagnosis of PD and the development of DPD in PD patients. The proposed method could further identify regions of interest in NDPD and DPD relevant to the disease onset such that physicians could perform an early diagnosis in time for available treatment. More importantly, our method performed well without any prior feature information that restricted analysis to only a few brain regions, demonstrating its ability to be executed by untrained operators and to be applied to unseen patient data for both the diagnosis of PD and the assessment of depression comorbidity.

## 2. Materials and Methods

### 2.1. Participants and Clinical Evaluation

This study was approved by the Medical Research Ethical Committee of Nanjing Brain Hospital (Nanjing, China) in accordance with the Declaration of Helsinki, and written informed consent was obtained from all subjects. A total of 276 PD patients, including 84 depressed PD (DPD) patients and 192 NDPD subjects, along with 200 healthy controls (HCs) were recruited. All the demographic characteristics and clinical symptom ratings were collected before MRI scanning, and all patients were in the ON state during the MRI scan. All subjects underwent a complete neurological and psychological status assessment and a review of medical history records. Mini-mental state examination (MMSE) was used to evaluate cognition. DPD patients were diagnosed with the Diagnostic and Statistical manual of Mental Disorders, Fifth Edition (DSM-V) criteria by an experienced psychiatrist. Afterwards, the severity of depression was quantified using the Hamilton Depression Scale (HAMD). Unified Parkinson’s Disease Rating Scale-Motor (UPDRS-III) was recorded for motor function, and Parkinson’s disease severity was rated according to Hoehn and Yahr (H & Y). The same metrics listed above except UPDRS-III and H & Y were applied to the control group. The neurocognitive tests were administered to each participant individually by a professional appraiser in the neuropsychological research center. The demographic and clinical data of patients with NDPD, DPD, and HC were compared using a Fisher’s exact test (for sex), multivariate analysis of variance (MANOVA) (for age, education, MMSE, and HAMD), and analysis of variance (ANOVA) (for UPDRS-III and H & Y between NDPD and DPD only). The level of significance was set at p<0.05 for standard comparison and at p<0.016 for multiple comparison with Bonferroni correction.

### 2.2. MRI Acquisition and Preprocessing

Images were scanned on Siemens verio 3.0T superconducting MRI system with 8-channel head coil in the department of radiology. The structural scans were acquired using 3D T1-Flair with the following parameters: repeat time (TR) = 2530 ms, echo time (TE) = 3.34 ms, matrix = 256, flip angle(FA) = $7^{\circ }$, thickness = 1.33 mm, gap = 0.5 mm, slices = 128. Resting-state BOLD-fMRI was collected axially using an echo-planar imaging (EPI) sequence with the following parameters: TR = 2000 ms, TE = 30 ms, FA = $90^{\circ }$, field of view (FOV) = 24 cm × 24 cm, matrix = 64 × 64, NEX = 1, slices = 31, thickness = 3.5 mm, gap = 0.6 mm. The subjects were instructed to keep their eyes closed, relax their minds and remain as motionless as possible during the data acquisition. Rubber earplugs were used to reduce noise, and foam cushioning was used to fix the head to reduce motion artifacts. The MR images were retrieved from the archive by two experienced neuroradiologists (Qingling Huang & Xiao Wang).

**Figure 1:**
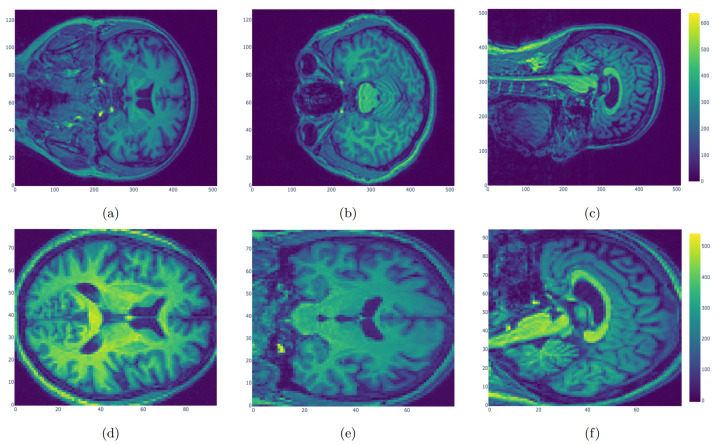
Original MRI images (**a**–**c**) and normalized MRI images (**d**–**f**). The original images of size (512, 512, 128) were normalized using Statistical Parametric Mapping. Individual images for all subjects were mapped to a common reference space with size (79, 95, 79) to reduce the complexity.

Three-dimensional T1-weighted images from both PD patients and HCs were then normalized using Statistical Parametric Mapping (https://www.fil.ion.ucl.ac.uk/spm/software/spm12/, accessed date: 20 August 2019) on the Matlab platform. The detailed step included spatial normalization to the Montreal Neurological Institute (MNI) space using the transformation parameters estimated via a unified segmentation algorithm [36] (Figure 1). In particular, the unified segmentation algorithm adopts a probabilistic framework that enables image registration, tissue classification, and bias correction to be combined within the same generative model. The procedure involves the minimization of a cost function that quantifies the differences between the individual image space and the template. Individual images for all subjects were therefore mapped from their individual MRI imaging space to a common reference space. As a result, the images of original size of (512, 512, 128) were converted into images of size (79, 95, 79). This meant passing from 33,554,432 to 592,895 voxels such that the complexity of the following analysis was dramatically reduced without loss of relevant information.

### 2.3. Multinomial Tensor Regression

The normalized 3D sMRI scans in our cases have 79×95×79 = 592,895 voxels, i.e., 592,895 parameters to be estimated in a regression setup, if each voxel is treated as a covariate. The authors in [34] proposed the family of tensor regression models that incorporate the special structure of tensor covariates encoded in these images for binary classification. The curse of dimensionality is diminished by imposing a low rank approximation to the extremely high-dimensional full coefficient array, which allows the development of a fast estimation algorithm and regularization. Adapted to our case, we propose the following multinomial tensor regression model to discriminate between NDPD, DPD, and HC.

Let *n* denote the generic sample size representing the number of 3D images exploited for fitting the model. Recall that we have a total of three categories. For 1≤i≤n,1≤j≤3, let $Y_{ij}$ be the binary variable that indicates whether the *i*th subject has NDPD, DPD, or this person is healthy. That is:

- $Y_{i1}=1$ if the *i*th patient is healthy and 0 otherwise;
- $Y_{i2}=1$ if the *i*th patient has DPD and 0 otherwise;
- $Y_{i3}=1$ if the *i*th patient has NDPD and 0 otherwise.

Denote $X_{i}\in R^{79\;\times \;95\;\times \;79}$ as the three-dimensional array encoded in the sMRI scan for the *i*th subject. The multinomial tensor classification model can be expressed as:

(1) $$\begin{array}{cc} \; & Y_{ij}∼\mathrm{Multinomial}\;\left(\mu_{i1},\mu_{i2},\mu_{i3}\right), \end{array}$$

(2) $$\begin{array}{cc} \; & \log\left(\frac{\mu_{i1}}{\mu_{i3}}\right)=\langle B_{1},X_{i}\rangle , \end{array}$$

(3) $$\begin{array}{cc} \; & \log\left(\frac{\mu_{i2}}{\mu_{i3}}\right)=\langle B_{2},X_{i}\rangle \end{array}$$

where $\langle B_{k},X_{i}\rangle$ represents the inner product of tensor $B_{k}$ and $X_{i}$ for k=1,2. $B_{k}\in R^{79\;\times \;95\;\times \;79}$ is a weight tensor in the form of

$$B_{k}=\sum_{r=1}^{R}\beta_{k,r}^{1}\circ \beta_{k,r}^{2}\circ \beta_{k,r}^{3},$$

i.e., a rank-*R* CP decomposition of $B_{k}$, where $\beta_{k,r}^{1}\in R^{79},\beta_{k,r}^{2}\in R^{95}$, and $\beta_{k,r}^{3}\in R^{79}$ are three vector components, and $\beta_{k,r}^{1}\circ \beta_{k,r}^{2}\circ \beta_{k,r}^{3}$ denotes their outer product. For our application, we use R=1 and simplify the notation by $B_{k}=\beta_{k}^{1}\circ \beta_{k}^{2}\circ \beta_{k}^{3}$ for the rest of the paper, but the framework applies to cases where R>1.

Similarly, we define $\beta_{k}^{1}\circ \beta_{k}^{2}$ as the outer product of $\beta_{k}^{1}$ and $\beta_{k}^{2}$. It is easy to see that each slice of $B_{k}$ on the first two dimensions equals the multiplication of $\beta_{k}^{1}\circ \beta_{k}^{2}$ by a scalar. In addition, each entry in $B_{1}$, $B_{2}$ reveals the log odds ratio of each voxel in the 3D image for either HC vs. NDPD or DPD vs. NDPD. Thus, by imposing the structure on coefficient matrices $B_{1},B_{2}$, the multinomial Models (1)–(3) have only 2×(79+95+79)=506 parameters, which is manageable given our sample size.

This model also allows for intercepts and 1-dimensional covariates, and it can be easily extended to cases with K>3 classes. A general form can be described as follows:

$$\begin{array}{cc} \; & Y_{ij}∼\mathrm{Multinomial}\;\left(\mu_{i1},\ldots ,\mu_{iK}\right),\\ \; & \log\left(\frac{\mu_{ik}}{\mu_{iK}}\right)=\alpha_{k}+\langle \gamma_{k},Z_{i}\rangle +\langle B_{k},X_{i}\rangle ,\;k=1,\ldots ,K-1 \end{array}$$

where $\alpha_{k}$ is an intercept, and $\gamma_{k}$ is the coefficient vector for the covariate vector $Z_{i}$. In practice, $Z_{i}$ contains characteristics including demographical and clinical traits for the *i*th subject.

### 2.4. Estimation

For the model specified in (1) to (3), we estimate the parameters by maximizing the likelihood. Given the observed imaging data $X_{i}$ and the binary indicators of the three classes $Y_{i1},Y_{i2},and\;Y_{i3}$ for i=1,…,n, the log-likelihood function can be expressed as:

(4) $$\begin{array}{c} l\left(\beta_{1}^{1},\beta_{1}^{2},\beta_{1}^{3},\beta_{2}^{1},\beta_{2}^{2},\beta_{2}^{3}\right)=\sum_{i=1}^{n}\sum_{k=1}^{3}y_{ik}\log\left(\mu_{ik}\right), \end{array}$$

where:

(5) $$\begin{array}{cc} \mu_{i1} & =\frac{\exp(\langle \beta_{1}^{1}\circ \beta_{1}^{2}\circ \beta_{1}^{3},X_{i}\rangle )}{1+\exp\left(\langle \beta_{1}^{1}\circ \beta_{1}^{2}\circ \beta_{1}^{3},X_{i}\rangle \right)+\exp\left(\langle \beta_{2}^{1}\circ \beta_{2}^{2}\circ \beta_{2}^{3},X_{i}\rangle \right)}, \end{array}$$

(6) $$\begin{array}{cc} \mu_{i2} & =\frac{\exp(\langle \beta_{2}^{1}\circ \beta_{2}^{2}\circ \beta_{2}^{3},X_{i}\rangle )}{1+\exp\left(\langle \beta_{1}^{1}\circ \beta_{1}^{2}\circ \beta_{1}^{3},X_{i}\rangle \right)+\exp\left(\langle \beta_{2}^{1}\circ \beta_{2}^{2}\circ \beta_{2}^{3},X_{i}\rangle \right)}, \end{array}$$

(7) $$\begin{array}{cc} \mu_{i3} & =\frac{1}{1+\exp\left(\langle \beta_{1}^{1}\circ \beta_{1}^{2}\circ \beta_{1}^{3},X_{i}\rangle \right)+\exp\left(\langle \beta_{2}^{1}\circ \beta_{2}^{2}\circ \beta_{2}^{3},X_{i}\rangle \right)}. \end{array}$$

The parameters $\Theta =\left(\beta_{1}^{1},\beta_{1}^{2},\beta_{1}^{3},\beta_{2}^{1},\beta_{2}^{2},\beta_{2}^{3}\right)$ are solved by block relaxation [37] as in Algorithm 1.
**Algorithm 1.** Block relaxation algorithm for maximizing (4). Initialize ${\left\{\beta_{k}^{j^{\left(0\right)}}\right\}}_{j=1,2,3;k=1,2}$ with random values **repeat**
(t≥1)  **for**
j=1,2,3 and k=1,2 **do**   $\beta_{k}^{j^{\left(t+1\right)}}={\mathrm{argmax}}_{\beta_{k}^{j}}l\left(\beta_{1}^{1^{\left(t+1\right)}},\beta_{1}^{2^{\left(t+1\right)}},\ldots ,\beta_{k}^{j},\ldots ,\beta_{3}^{2^{\left(t\right)}}\right)$  **end for** **until**
$|l\left(\Theta^{\left(t+1\right)})-l(\Theta^{\left(t\right)}\right)|<\epsilon$

The parameters are updated in a blockwise manner until convergence. When block relaxation is used for binary classification as in [34], the subproblem for $\beta_{k}^{j}$ can be reduced to a general logistic regression setup since the inner product term $\langle \beta_{k}^{1}\circ \beta_{k}^{2}\circ \beta_{k}^{3},X_{i}\rangle$ can be transformed into $\langle \beta_{k}^{j},X_{i(j)}\left(\beta_{k}^{1}⊙\beta_{k}^{2}⊙\cdots ⊙\beta_{k}^{j-1}⊙\beta_{k}^{j+1}⊙\cdots ⊙\beta_{2}^{3}\right)\rangle$, where ⊙ denotes the Khatri–Rao product. However, the same technique does not apply to multinomial tensor regression as the likelihood becomes much more complicated. Therefore, we adopt Adam optimizer [38] for solving the subproblems in Algorithm 1. The above estimation procedure was implemented in Python TensorFlow.

## 3. Results

### 3.1. Clinical and Demographic Data

In Table 1, we provided the complete demographic and clinical information for all subjects participating in this study. No significant difference was observed between the gender, ages, education, and MMSE and UPDRS-III scores of PD including DPD and NDPD patients in comparison to the HCs, while significant differences were detected with respect to the HAMD and H & Y scores among the three groups. In particular, for DPD patients, the HAMD scores (20.2±4.6) were significantly higher than those for NDPD patients (6.9±3.1) and HCs (2.2±2.3), while the H & Y scales (1.4±0.6) were significantly lower than those for NDPD patients (1.8±0.7). Our goal is to develop a tensor regression model that performs well without any prior medical information. Since the HAMD score is the most widely used clinician-administered scale for assessing depression and the H & Y scale is used to measure how Parkinson’s symptoms develop, both of which are strongly correlated with the progression of PD and DPD, we did not include these two metrics when building the tensor regression model.

### 3.2. Quantitative Performance

The performance of our tensor regression Model (1) to (3) was evaluated and tested on NDPD and DPD patients and healthy controls. The sMRI scans for all subjects were first randomly divided into training set (80%) and testing set (20%) while retaining the DPD:NDPD:HC ratio in both sets. We first obtained the parameter estimates using the method specified previously based on the training set. The learning rate in the Adam optimizer was tuned through 10-fold cross-validation. Next, we evaluated the fitting performance and prediction performance on the training and testing sets, respectively.

We compared the performance of the proposed method with the multinomial logistic regression as well as 3D CNN, where each voxel was treated as a covariate. Note that the multinomial logistic regression could not handle the large dimension of over 500,000. Therefore, we only considered taking the first 1000, 3000, and 10,000 voxels as predictors and built the multinomial logistic regression models under these three cases. The multinomial logistic regression model was implemented in R glmnet, and 3D CNN was built in Python deep learning API keras. The area under the ROC curve (AUC) is a widely used measure of performance of supervised classification rules. However, the simple form is only applicable to the case of two classes. We adopted the AUC calculation for the case of more than two classes by averaging pairwise comparisons [39], i.e., the multi-class extension of the AUC approach (MAUC). In particular, this measure reduces to the standard form in the two-class case. In addition to MAUC, we also evaluated model performance on both the training and testing sets using Prediction Accuracy (PA) and Rand Index (RI). PA was calculated through number of correct classifications divided by the sample size, and the metric of RI was computed following the definition in [40].

The results were provided in Table 2 and Table 3. We found that the proposed method achieved a perfect fitting accuracy in the training set and the highest prediction accuracy in the testing set among all the competitors. The performance of 3D CNN on the training set is superior, while its performance on the testing set is much worse compared with that on the training set, which is understandable due to the problem of possible over-fitting. Furthermore, based on the MAUC values, our method was shown to be quite robust with respect to the varying thresholds.

### 3.3. Aberrant Structural Brain Regions

**Figure 2:**
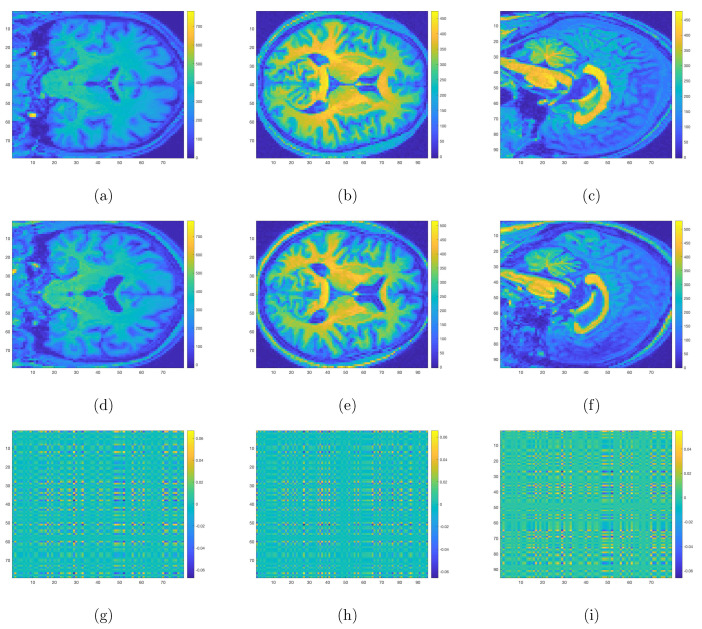
Three-dimensional sMRI images for NDPD (**a**–**c**), HC (**d**–**f**), and the heatmaps for coefficient matrices corresponding to three different surfaces respectively (**g**–**i**). Voxels with yellow and dark blue colors correspond to regions with aberrant structural changes for NDPD compared with HC.

One major advantage of our method was that one could simultaneously reveal the most discriminative structural changes in NDPD and DPD patients. In Figure 2, we drew the heatmaps for the coefficient matrices $\beta_{1}^{1}\circ \beta_{1}^{2},\beta_{1}^{1}\circ \beta_{1}^{3}$ and $\beta_{1}^{2}\circ \beta_{1}^{3}$ in Model (2) corresponding to three different surfaces and aligned the locations with significant values to the sMRI images for both NDPD patients and healthy subjects. Specifically, the disease-related alterations were found mainly in the bilateral frontotemporal and occipital lobes, basal ganglia, thalamus, corpus callosum, midbrain, and cerebellum.

**Figure 3:**
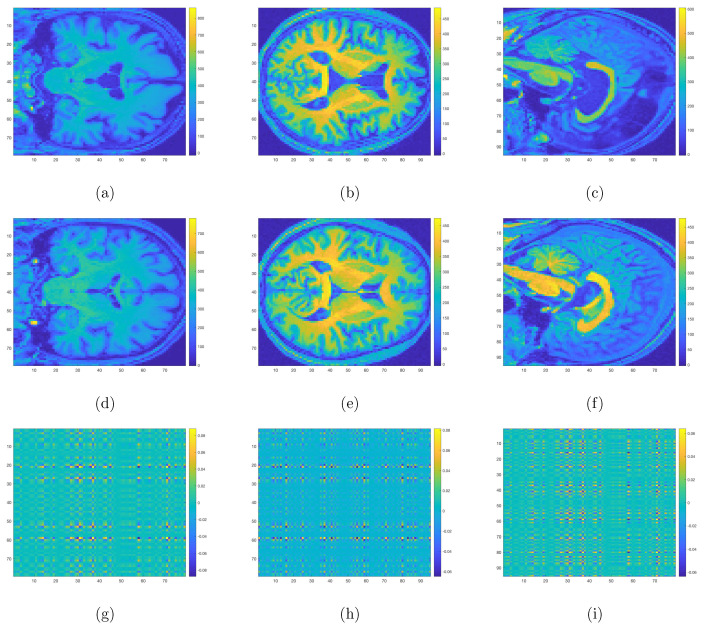
Three-dimensional sMRI images for DPD (**a**–**c**), NDPD (**d**–**f**), and the heatmaps for coefficient matrices corresponding to three different surfaces respectively (**g**–**i**). Voxels with yellow and dark blue colors correspond to regions with aberrant structural changes for DPD compared with NDPD.

Figure 3 allows us to structurally visualize the differences in DPD and NDPD by plotting the coefficient matrices $\beta_{2}^{1}\circ \beta_{2}^{2},\beta_{2}^{1}\circ \beta_{2}^{3}$, and $\beta_{2}^{2}\circ \beta_{2}^{3}$ in Model (3). In particular, compared with NDPD, the DPD group displayed distinguishable differences in the corpus callosum, the cerebellum, and the right superior temporal gyrus. At the same time, the bilateral fronto-occipital lobe, left temporal lobe, bilateral basal ganglia, and thalamus also showed significant differences.

## 4. Discussion

In this study, we built and validated a multinomial tensor-regression-based framework that leveraged 3D sMRI scans to simultaneously differentiate among NDPD, DPD, and healthy subjects. Other than the tool of tensor regression, interested readers might be aware of another class of machine-learning-based methods to localize PD in the brain (i.e., localization of disease biomarkers). Salvatore et al. [41], Zhang et al. [42], Abós et al. [43], Palumbo et al. [44] used machine learning algorithms based on either principal components analysis (PCA) or Support Vector Machine (SVM) that allowed individual differential diagnosis of PD to obtain voxel-based morphological biomarkers of PD. Another school of research including [45,46,47] focused on Region of Interest methods (ROI), where some specific regions of the brain such as the gray matter and hippocampal volume were extracted due to a priori knowledge about their effects on brain functionality and memory. More recently, a computer-based technique utilizing CNNs [31,32,33] to create prognostic and diagnostic biomarkers has been more widely adopted and has generated a lot of attention. These methods also exploited 3D structural MRI and required no prior knowledge on significant regions that might impact the progress of PD. However, these CNN-based methods usually require large memory and extensive computation time. For example, the average run time of fitting a tensor regression model to the 3D normalized MRI images was about 1586 s, and the processor was a 2.3 GHz dual-core Intel Core i5 with 8 GB memory, while the typical run time for fitting a CNN model was about more than 10 times of 1586 s on the same processor. Hence, compared with the tensor-regression-based method, implementing a CNN tends to be more computationally expensive. Furthermore, the intuitions behind these CNN-based methods are not as straightforward as tensor regression in the sense that not only could the coefficients for CNN not be explicitly interpreted as in Figure 2 and Figure 3, but the underlying theoretical property for CNN is also still yet to be justified, while for tensor regression, the complete convergence analysis has been thoroughly investigated [34,35].

The proposed method certainly has limitations. First, this method was only based on the tensor regression method with structural MRI rather than the previous methods based on network and functional MRI, which would be excellent topics for future studies. Second, the sample size in typical neuroimaging studies, including the current study, is quite small compared to the large image voxel size. Hence, the high-dimensional challenge remains pervasive rather than an exception in neuroimaging analysis. In these cases, regularization becomes essential for stabilizing the coefficient estimates and for minimizing the harm of over-fitting. In the near future, for sample-starved studies, we intend to either use penalty regularization [34,48] or impose sparsity through multiway shrinkage priors [49] for identifying sub-regions associated with the PD. We will also include more subjects to diminish the threats caused by the high dimensionality. Furthermore, we will consider fitting the multinomial tensor regression with R≥2 and compare its performance with the current findings. The ultimate goal is to build a tensor regression model that integrates both structural MRI and functional MRI and can accommodate the high-dimensional nature of imaging data. This requires an original and creative combination of knowledge and tools from high-dimensional statistics, radiomic analysis, and biostatistics focusing on radiological studies. The proposed research will thus advance knowledge at the crossroads of several exciting fields of statistics and bioinformatics. The proposed work is of potentially transformative nature by substantially broadening the paradigm of inference of neuroimaging data for disease risk stratification using traditional classification methods and extending the tensor regresssion methods to substantially new and complex domains.

## 5. Conclusions

To the best of our knowledge, this study was the first attempt to construct a tensor-regression-based platform for structurally discriminating among NDPD, DPD, and HC with a high prediction accuracy. In terms of clinical characteristics, significant differences were detected with respect to the HAMD and H & Y scores among three groups of DPD, NDPD, and HC. In terms of regions with abnormal structures, significant differences were found in bilateral the frontotemporal and occipital lobes, basal ganglia, thalamus, corpus callosum, midbrain, and cerebellum between NDPD and HC. Concurrently, structural differences in the corpus callosum, cerebellum, and the right superior temporal gyrus, as well as the bilateral fronto-occipital lobe, left temporal lobe, bilateral basal ganglia, and thalamus were detected between DPD and NDPD.

These findings suggest disease-related alterations of structure as the basis for faulty information processing in this disorder. Our findings were in good agreement with the alternative structure and functional features in cortical regions, cerebellum, brainstem, bilateral basal ganglia, thalamus, and limbic regions in previous studies [26,27,28,29,50,51]. More importantly, our algorithm performed well without any prior feature information and regardless the variability of imaging protocols and scanners, demonstrating its feasibility to be executed by untrained operators and to be generalizable to unseen patient data to support the diagnosis of both PD and the progression of DPD. In conclusion, tensor regression facilitates a deeper understanding into the abnormalities in DPD and PD and plays an essential role in statistical analysis of high-dimensional complex MRI imaging data to support the radiological diagnosis of comorbidity of depression with PD.

## Figures and Tables

**Table 1:** Clinical and demographic data evaluation of NDPD, DPD, and HC. *^a^*. The *p* value for gender distribution by Fisher’s exact test. *^b^*. The *p* value for age by multivariate analysis of variance (MANOVA). *^c^*. The *p* value for education by MANOVA. *^d^*. The *F* test statistic and the *p* value for MMSE scores by MANOVA. *^e^*–*^g^*. The *p* values for HAMD scores by Paired-Samples *t* test with Bonferroni correction for further comparison between three groups. *^h^*. The *F* test statistic and the *p* value for UPDRS-III by analysis of variance (ANOVA). *^i^*. The *F* test statistic and the *p* value for H & Y by ANOVA.

Characteristics	DPD (*n* = 84)	NDPD (*n* = 192)	HC (*n* = 200)	Test Statistic	*p* Value
Sex (M/F)	36/48	104/88	96/104	0.409	>0.05 ${}^{a}$
Age (year)	58.1±7.5	57.8±7.0	57.8±5.5	0.021	>0.05 ${}^{b}$
Education (year)	11.0±3.1	11.8±3.3	11.7±4.8	0.689	>0.05 ${}^{c}$
MMSE	28.7±1.1	28.6±1.7	29.0±2.3	0.585	>0.05 ${}^{d}$
HAMD	20.2±4.6	6.9±3.1	2.2±2.3	243.2 (p<0.05)	<0.016 ${}^{e}<0.016$ ${}^{f}<0.016$ ${}^{g}$
UPDRS-III	28.3±13.2	26.4±13.3	N/A	0.295	>0.05 ${}^{h}$
H & Y	1.4±0.6	1.8±0.7	N/A	5.37	<0.05 ${}^{i}$

**Table 2:** The summary statistics for prediction performance on the training set for all methods.

Model	RI	PA	MAUC
Multinomial Tensor	1	1	1
Multinomial Logistic (d=1000)	0.59	0.61	0.69
Multinomial Logistic (d=3000)	0.6	0.63	0.64
Multinomial Logistic (d=10,000)	0.66	0.68	0.73
3D CNN	1	1	1

**Table 3:** The summary statistics for prediction performance on the testing set for all methods.

Model	RI	PA	MAUC
Multinomial Tensor	0.89	0.94	0.98
Multinomial Logistic (d=1000)	0.49	0.44	0.55
Multinomial Logistic (d=3000)	0.56	0.56	0.69
Multinomial Logistic (d=10,000)	0.58	0.63	0.70
3D CNN	0.55	0.31	0.53

## Data Availability

The datasets used and/or analyzed during the current study are available from the corresponding author on reasonable request. The data are not publicly available due to privacy or ethical restrictions.
